# Nanosecond Infrared Laser Sampling of Mouse and Human Liver Tissues for LC-MS/MS Analysis of Bile Acids

**DOI:** 10.3390/ijms27104572

**Published:** 2026-05-20

**Authors:** Marceline M. Fuh, Jan Hahn, Markus Heine, Ioannis Evangelakos, Ludger Scheja, Oliver Mann, Anna Worthmann, Hartmut Schlüter, Joerg Heeren

**Affiliations:** 1 Department of Biochemistry and Molecular Cell Biology, University Medical Center Hamburg-Eppendorf, 20246 Hamburg, Germany; 2 Section Mass Spectrometry and Proteomics, University Medical Center Hamburg-Eppendorf, 20246 Hamburg, Germany; 3 Department of General, Visceral and Thoracic Surgery, University Medical Center Hamburg-Eppendorf, 20246 Hamburg, Germany

**Keywords:** bile acids, tissue homogenization, nanosecond infrared laser, laser ablation, mass spectrometry

## Abstract

Accurate quantification of bile acids is vital as they are potential biomarkers for several diseases. To solubilize bile acids from tissues, homogenization is required, typically performed using mechanical methods such as the TissueLyser method. Drawbacks like available tissue amount and low abundance of bile acids interfere with the analysis. We aim to establish a nanosecond infrared laser (NIRL) as a possible tissue homogenization tool to circumvent problems associated with limited sample amounts for research and to target low abundance bile acids. We quantify bile acids from mouse and human liver tissues comparing both the classic TissueLyser and the NIRL methods for homogenization prior to LC-MS/MS analysis. The applicability of our approach is tested using mice lacking Cyp2c70, a well-established mouse model with an altered bile acid composition compared to wild type mice. We found the NIRL method to require an almost 14 times smaller starting tissue amount than the TissueLyser method. The NIRL is also comparable to the TissueLyser for high abundance bile acids. While the TissueLyser provides efficient mechanical homogenization, the NIRL potentially enables quantification of low abundance bile acids. The well-established biological differences in bile acid profiles from the Cyp2c70 knockout mouse model were also observed with NIRL homogenization. These results show that the NIRL is potentially useful as smaller tissue amounts are required for analysis and low abundance bile acids are quantifiable.

## 1. Introduction

Bile acids are a class of structurally diverse amphiphilic molecules that are products of cholesterol catabolism in hepatocytes (primary bile acids) and further heavily modified in the lower gastrointestinal tract to produce a broad range of secondary bile acids [1]. Their history of elucidation, structure–function relationship, analytical methods and metabolism have been extensively reviewed by Hoffmann and Hagey [2]. Biosynthesis of bile acids occurs either via the classical/neutral pathway initiated by cholesterol 7α-hydroxylase (CYP7A1) or via the alternative/acidic pathway, which can be initiated by different enzymes that are also expressed outside the liver [3,4]. With majority of bile acid knowledge stemming from experiments with mice, it is important to note that in contrast to humans, about half of the bile acid pool in mice consists of 6-hydroxylated muricholic acids (MCAs), and Cyp2c70 has been shown to be responsible for these differences. In addition, the primary bile acids in humans (cholic acid (CA) and chenodeoxycholic acid (CDCA)) are conjugated with the amino acid glycine, while those in mice (CA and muricholic acid (MCA)) are conjugated with taurine [4,5,6].

Bile acids are generally responsible for the production of bile, intestinal absorption of dietary lipids and proteolytic cleavage of dietary proteins, antimicrobial activities in the small intestine, and cholesterol homeostasis [3]. They have also been shown to be involved in various metabolic diseases and therapies [7,8,9]. Some bile acids, like ursodeoxycholic acid (UDCA), are being used as a medication for the treatment of cholestatic liver diseases, and CDCA has been used since the early 1970s for bowel dysfunctions in irritable bowel disease and was approved as an orphan indication for the treatment of patients with cerebrotendinous xanthomatosis (CTX) [10,11,12].

The analysis of bile acids has greatly advanced from the use of M. von Pettenkofer’s color reaction in the 1800s to mass spectrometry based methods for identification and quantification today [13]. Depending on several factors, including volatility, sample size, time and availability of analytical device, the method of choice usually varies between gas chromatography mass spectrometry (GCMS); liquid chromatography mass spectrometry (LCMS); nuclear magnetic resonance spectroscopy (NMR); or supercritical fluid chromatography (SFC-MS) [13,14]. Analysis of bile acids with mass spectrometry from biological tissues and fluids usually involves a sample extraction step prior to mass spectrometry. To extract these biomolecules from the biological matrices, a tissue homogenization step is required. This usually requires the use of mechanical methods involving tools and instruments or non-mechanical homogenization methods utilizing lysis buffers, detergents and temperature [15,16]. Whilst these methods and tools are widely available, each has its pros and cons that should be evaluated based on sample and experimental specific requirements. Mechanical homogenization is particularly used for tissues and collagen rich biological matrices, which is typically applied through liquid nitrogen grinding with mortar-and-pestle, ball mills or vortexer bead beating [15,17]. These methods have drawbacks such as: requiring large initial tissue amounts, a low recovery rate, the presence of large particles like cell debris, low throughput and insoluble compounds that require additional steps to be removed [18].

Over the past decades, laser interaction with biological tissues have been extensively studied and heavily applied in the field of medicine [19]. Once a laser beam is produced and aimed at a biological tissue, four basic interactions are possible: reflection, transmission, scattering or absorption. For tissue ablation to occur with the generation of an aerosol, the exploited laser–tissue interaction is usually absorption. In 2015, Kwiatkowski et al. demonstrated the ability to softly release and solubilize proteins from tissues via cold vaporization with the aerosol of a picosecond infrared laser (PIRL), without fragmenting the proteins [20]. Since then, ultrashort pulsed infrared lasers, tuned to a wavelength of 2950 nm, targeting a peak in the absorption spectrum of water have been used to completely homogenize biological tissues prior to biomolecular analysis [21,22,23]. Other pulsed lasers such as a microsecond infrared laser (MIRL) have been used for proteomics [24] and recently a nanosecond infrared laser (NIRL) has been used for proteomics [25,26,27] and lipidomics [28]. Based on the intended study, the choice of laser technology used is very crucial. Some lasers, like a MIRL, transfer significant amounts of thermal energy to the surrounding ablation zone, leading to cooking/burning of the tissue. However, by using ultrashort pulses in the picosecond- or nanosecond-regime there is no heat transfer into the tissue.

In this study, we explore the possibility of using very small amounts of samples at the start of experiments to obtain biologically relevant data. For the first time, we use the NIRL to investigate its potential for liver tissue homogenization from mice and humans by analyzing the generated aerosols for bile acid quantification using liquid chromatography and mass spectrometry (LC-MS/MS). This method is also compared to the classical mechanical tissue homogenization method usually employed in most research experiments for tissue homogenization.

## 2. Results

**Figure 1:**
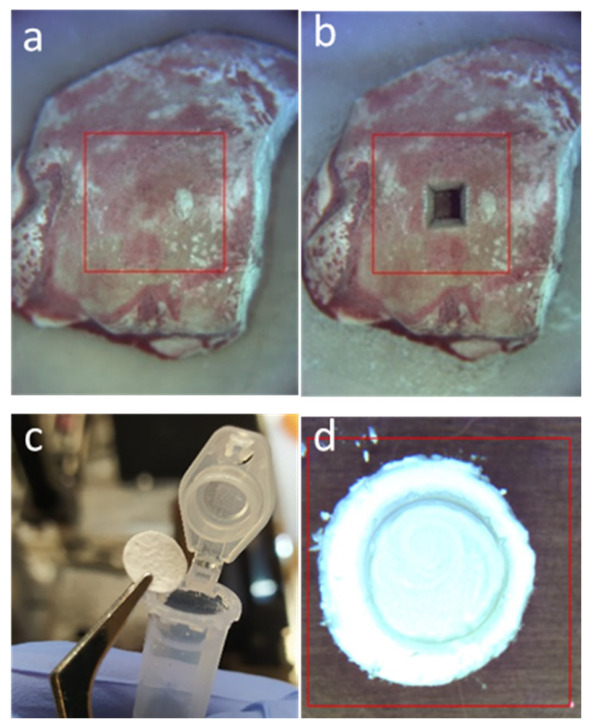
(**a**,**b**) Example images of freshly frozen mouse liver tissue before ablation and after ablation. (**c**) Image of glass fiber filter prior to aerosol collection. (**d**) Example of condensed NIRL ablated mouse liver aerosol on a glass fiber filter paper. Red square indicates the region of interest.

To investigate the eligibility of NIRL based ablation for tissue sampling and homogenization prior to bile acid quantification, freshly frozen liver tissues obtained from both mice and humans are ablated and the generated aerosols are collected and processed using liquid chromatography mass spectrometry. Example images of freshly frozen liver tissues before and after ablation and the collected condensed aerosol are as pictured in Figure 1.

**Figure 2:**
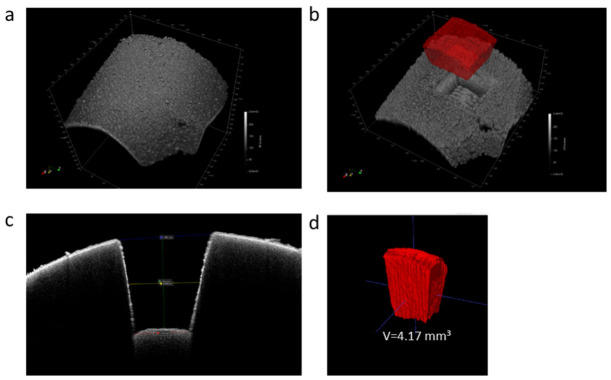
Example 3D OCT scan images of mouse liver tissues (**a**) before ablation and (**b**) after the ablation process with a 3D representation of the ablated volume (red) in corrected aspect ratios. Determination of the dimensions of the ablation site in (**c**) a OCT brightness scan (B-scan) and (**d**) the 3D rendering of the manually segmented volume of the ablation in the original OCT resolution (axial: 3.45 µm, lateral: 8.23 µm).

Optical coherence tomography (OCT) imaging is used to acquire the 3D surface of the liver tissues before and after ablation to reconstruct and quantify the ablated tissue volume and calculate the weight of the NIRL homogenized tissue for data normalization. Figure 2 illustrates example 3D OCT scan images of a mouse liver tissue before and after ablation. The ablated volume is calculated; the depth is d = 1.08 mm and the width is measured in three different depths and determined to be w1 = 2.05 mm, w2 = 1.88 mm and w3 = 1.57 mm (Figure 2c). The ablation volume is quantified to 4.17 mm^3^ based in manual segmentation.

**Figure 3:**
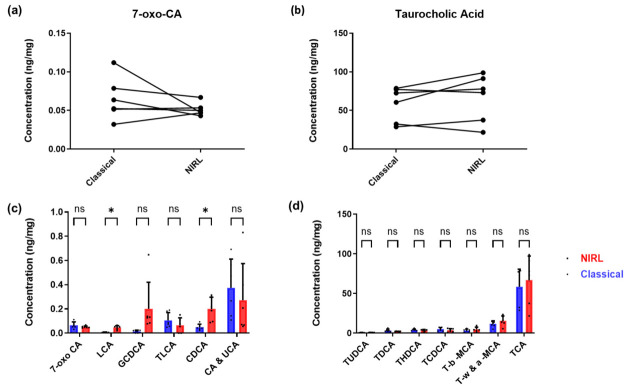
(**a**,**b**) Representative plots of bile acid profiles of the least abundant (7-oxo-CA) and most abundant (taurocholic acid) bile acids, showing individual liver tissues homogenized either classically or with the NIRL. Each liver tissue piece, which was halved, is matched to the corresponding homogenization method. (**c**,**d**) Average concentrations in ng/mg of bile acid species in mouse tissues that were either classically homogenized (*n* = 6) or ablated with the NIRL (*n* = 6). Error bars indicate standard deviation (SD). Statistical analysis was performed using a Student’s T-Test. * *p* < 0.05. ns stands for not significant.

To understand the possible applicability of NIRL sampling prior to bile acid quantification, every mouse liver is divided in half; one part is sampled with the laser and the other half with the classical TissueLyser. These mouse liver tissues are obtained from mice housed at different temperatures (6 °C and 30 °C) that are either ablated using the NIRL (*n* = 6) or classically homogenized using the TissueLyser (*n* = 6). Bile acids from both methods are quantified using LC-MS/MS. Figure 3 shows the concentrations of different bile acids normalized per milligram tissue (Supplementary Table S1) quantified from both the classical and laser approaches.

**Figure 4:**
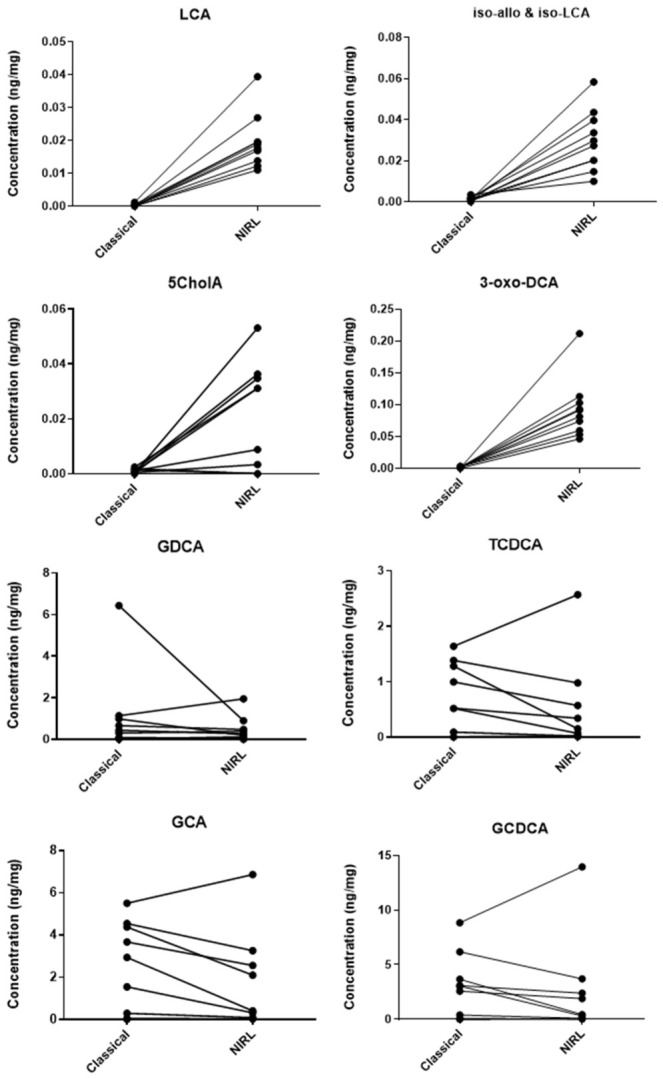
Plots of different bile acids from each human liver tissue sample classically homogenized with the TissueLyser or ablated with the NIRL. Each liver tissue piece, which was halved, is matched to the corresponding homogenization method (*n* = 10).

For application with patient samples, 10 human liver tissues obtained after biopsy during bariatric surgery were ablated with the NIRL and the corresponding piece homogenized with the TissueLyser. The relationships between the quantified bile acids from both approaches are shown in Figure 4, using the concentrations of different bile acids that have been normalized per milligram tissue (Supplementary Table S2). A Bland–Altman analysis was performed using an example of a low abundance bile acid (5cholA) and a high abundance bile acid (TCDCA) and is shown in Supplementary Figure S1. The analysis shows the NIRL demonstrates low-range sensitivity compared to the classical TissueLyser by resolving micro-concentrations and avoiding analytical floor effects across both low and high abundance bile acids. However, the validation is strictly limited by a small cohort size, which prevents a definitive calculation of the true population limits of agreement due to unstable sample variances.

**Figure 5:**
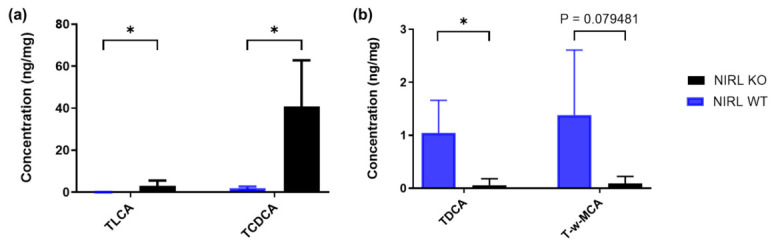
Quantification of bile acids in liver tissue aerosols of wild type mice (NIRL WT) in averaged replicates (*n* = 5 mice) compared to Cyp2c70 knockout mice (NIRL KO) in averaged replicates (*n* = 5 mice) ablated with the NIRL. (**a**) TLCA and TCDCA with lower concentrations in livers of NIRL WT mice. (**b**) TDCA and T-w-MCA with lower concentrations in livers of NIRL KO mice. Error bars indicate standard deviation (SD). Statistical analysis was performed using a Student’s T-Test. * *p* < 0.05.

To investigate if well-known and established biological differences can also be observed with bile acids quantified from NIRL aerosol, livers obtained from a Cyp2c70 knockout mouse model and wild type mice were homogenized with the NIRL and analyzed with LC-MS/MS. Figure 5 shows different bile acid concentrations that are either higher or lower in the wild type (*n* = 5) or knockout (*n* = 5) liver tissues (Supplementary Table S3). As expected, TLCA and TCDCA are lower in wild type mice compared to Cyp2c70 knockout mice (Figure 5a), as Cyp2c70 converts CDCA to MCA. In line, LCA, which is a secondary bile acid derived from CDCA, is expected to be higher in the Cyp2c70 knockout mice. On the other hand, TDCA and T-w-MCA are higher in wild type mice compared to Cyp2c70 knockout (Figure 5b). DCA is a secondary bile acid derived from CA, which is expected to increase in the wild type mice.

## 3. Discussion

In this study, freshly frozen liver tissues obtained from mice housed at different temperature conditions were used to establish the applicability of NIRL condensed aerosol for bile acid quantification using mass spectrometry. Then, freshly frozen human liver tissues obtained after liver biopsy from patients during bariatric surgery were also sampled with the NIRL and their bile acid profiles studied in comparison to the corresponding classically homogenized piece. Finally, freshly frozen liver tissues from a Cyp2c70 knockout mouse model and wild type were ablated with the NIRL to observe the known biological changes expected in the bile acid profiles.

Pictures of an example piece of liver tissue before and after laser ablation are shown in Figure 1. The ablation plume collected on a glass fiber filter paper can be seen as a condensed powder. Using an OCT imaging technique, a volume of approximately 4.2 µL is calculated to have been ablated with the NIRL. Given that the laser parameters are constant, the ablation process is very consistent, as equal volumes are obtained from ablating each freshly frozen liver tissue. As the density of mouse liver tissue is ~1.06 g/cm^3^ [29], the calculated weight of ablated tissue is ~4.452 mg. For classical homogenization using a TissueLyser or mortar/pestle, many studies start with an initial liver weight typically between 50 mg and 150 mg for bile acid quantification using LC-MS/MS [30,31,32]. In this study, liver samples homogenized with the TissueLyser have a start weight between 70 mg and 100 mg. This indicates that NIRL homogenization allows LC-MS/MS bile acid quantification with an average of 14–20 times smaller liver tissue amounts in comparison to classical homogenization techniques. This therefore suggests that NIRL ablation can be potentially useful in interdisciplinary research studies involving human biopsies, as more often than not there is a restriction to the number of analytical techniques that can be carried out with one tissue biopsy due to limitations in size. Other studies have also shown that smaller sample amounts generated from NIRL tissue sampling can be used to efficiently quantify other biomolecules, such as proteins and lipids, using mass spectrometry [25,28].

To study the possible differences on the bile acid profiles of NIRL sampling in comparison to TissueLyser homogenization, every mouse liver was divided in half; one part was sampled with the laser and the other with the classical TissueLyser, as can be seen in Figure 3. This is to reduce, as much as possible, the biological differences occurring due to mouse-to-mouse variations. Figure 3a,b illustrates examples of the quantified bile acids, with 7-oxo-CA representing a low abundance and TCA representing a high abundance bile acid. The amounts quantified from these individual liver samples, which were cut in half and either processed classically or with the laser, are on average within the same range. Figure 3c,d also shows the average bile acid profiles within six biological replicates each of classically homogenized and laser ablated tissues. These concentrations are also quite similar, with a majority of the bile acids showing no significant statistical differences between both methods. However, LCA and CDCA in the low abundance profile show a statistical difference, with the NIRL ablated samples demonstrating higher concentrations. This indicates that both NIRL ablation and TissueLyser homogenization generate similar bile acid amounts of the various bile acids quantified with NIRL being potentially advantageous at smaller ranges. Standard deviations within the sampling methods can be attributed to biological replicates. The slight differences in the amount of quantified bile acids between the NIRL and the classical method can be attributed to the heterogeneity of the liver. Although the liver is generally referred to as homogeneous due to its uniform histological appearance, it has long been established as a heterogeneous tissue with metabolic zones [33]. This is a problem that is generally faced in tissue biopsies, as access to an entire tissue is hardly possible for analysis [34,35]. These results therefore demonstrate the possible applicability of NIRL generated aerosol for bile acid quantification using mass spectrometry.

For NIRL application on bile acid quantification from patient samples, 10 human liver tissues obtained after biopsy were ablated with the NIRL and the corresponding piece homogenized with the TissueLyser. These patients comprised males and females, varying steatosis grades, fibrosis and type 2 diabetes mellitus. Figure 4 shows plots of different bile acids from each human liver tissue sample classically homogenized with the TissueLyser and ablated with the NIRL. Bile acids with low concentrations like LCA; iso allo LCA and iso LCA; 5CholA; and 3-oxo-DCA are almost undetectable in many patient samples when homogenized with the TissueLyser compared to the NIRL. This could be due to the fact that bead mill homogenizers such as the TissueLyser use collisions between beads to homogenize the sample, which may also leave small amounts of microscopic particulate matter, leading to a lower yield [18]. Owing to the complexity of the microbiome and the huge dynamic range of bile acids, low abundance bile acids can therefore be poorly extracted. Higher abundance bile acid profiles like those from GDCA; TCDCA; GCA; and CDCA, however, show concentrations in a similar range between both methods. This therefore indicates that the NIRL is potentially advantageous in extracting bile acids present in lower concentrations from tissues.

To investigate if well-known and established biological differences can also be observed with bile acids quantified from NIRL aerosol, livers obtained from wild type and the Cyp2c70 knockout mouse model were analyzed. CYP2c70 has been shown as responsible for the bile acid species differences between mice and humans, as it hydrolysis CDCA generated from both the classical and alternative pathways into the various muricholic acid species and their taurine conjugates in mice [3,6]. Therefore, in a Cyp2c70 knockout mouse model, all muricholic acid species and their taurine conjugates that are downstream from CYP2c70 activity should be significantly lower when compared to wild type mice. Consequently, higher levels of CDCA and its taurine conjugates should be observed in the Cyp2c70 knockout mouse model compared to wild type mice. This therefore indicates that known and expected biological changes of bile acids are observed in the aerosols of mouse liver ablated with NIRL prior to mass spectrometry.

This study presents certain limitations, primarily regarding its small cohort sizes, which limits the generalizability of the findings and restricts the statistical power required to definitively establish population-level limits of agreement in the Bland–Altman analysis. Additionally, estimating ablated tissue mass using OCT volumes and theoretical tissue density introduces inherent biological variability between replicates, though this confounding factor similarly impacts classical mechanical homogenization. Methodologically, the study did not evaluate analytical parameters such as the limit of detection (LOD), limit of quantification (LOQ), extraction recovery or matrix effects between the NIRL and TissueLyser methods. Finally, the use of the NIRL technique is constrained by distinct operational barriers like the high cost of laser instrumentation, specialized technical expertise required for operation, and a lower sample throughput.

In summary, we have shown for the first time that nanosecond infrared laser ablation of liver tissues from mice and human freshly frozen liver samples prior to bile acid quantification with LC-MS/MS generates similar results as compared to homogenization with the classical mechanical homogenization (for advantages and disadvantages of the technique see Supplementary Table S4). Known biological differences such as bile acid profiles between Cyp2c70 wild type and knockout mice can also be observed from the laser ablated tissue aerosol. As this laser technique successfully analyzes bile acids from very small initial sample material, which is about 14 times smaller than that required for classical tissue homogenization; this makes it particularly suitable for highly sought after human patient biopsies.

## 4. Materials and Methods

### 4.1. Bile Acid Analytical Standards and Solvents

Lithocholic acid (LCA), taurolithocholic acid-3 sufate (TLCA-3S), taurolithocholic acid (TLCA), cholic acid (CA), ursocholic aicd (UCA), 3-oxo-deoxycholic acid (3-oxo-DCA), 7-ketodeoxycholic acid (7-oxo-CA), tauroursodeoxycholic acid (TUDCA), taurohyodeoxycholic acid (THDCA), taurocholic acid (TCA), glycocholic acid (GCA), taurochenodeoxycholic acid (TCDCA), taurodeoxycholic acid (TDCA), glycochenodeoxycholic acid (GCDCA), glycodeoxycholic acid (GDCA), chenodeoxycholic acid (CDCA), glycolithocholic acid (GLCA), deoxycholic acid (DCA), glycolithocholic acid (GLCA), glycolithocholic acid-3 sulfate (GLCA-3S); 5-cholenic acid (5CholA), isodeoxycholic acid (isoDCA), isoallolithochlic acid (isoalloLCA); isoLithocholic acid (isoLCA), omega muricholic acid (wMCA), alpha muricholic acid (aMCA), beta muricholic acid (bMCA), tauro-omega muricholic acid (TwMCA), tauro-alpha muricholic acid (TaMCA), tauro-beta muricholic acid (TbMCA), lithocholic acid-d4 (d4-LCA), taurocholic acid-d4 (d4-TCA), cholic acid-d4 (d4-CA), chenodeoxycholic acid-d4 (d4-CDCA), deoxycholic acid-d4 (d4-DCA) and glycochenodeoxycholic-d4 acid (d4-GCDCA) were purchased from Sigma–Aldrich (Darmstadt, Germany), Steraloids Inc. (Newport, RI, USA), Isosciences LLC (Ambler, PA, USA) or Cayman Chemical (Ann Arbor, MI, USA). LC/MS grade methanol (≥99.9%), acetonitrile (≥99.9%), water, isopropanol (≥99.9%), ammonium acetate (≥99.99%) and formic acid (98–100%) were purchased from Merck (Darmstadt, Germany) and Sigma–Aldrich (Taufkirchen, Germany).

### 4.2. Calibration Curve and Internal Standard Preparation

A calibration stock solution was prepared containing all analytical bile acid standards with each having a final concentration of 30 µM. A 13-point calibration was then prepared with the following concentrations: 0.6 nM, 1.8 nM, 3 nM, 6 nM, 18 nM, 30 nM, 60 nM, 180 nM, 300 nM, 600 nM, 1.8 µM, 3 µM and 6 µM in methanol/acetonitrile (1/3, *v*/*v*), 20 mM ammonium acetate and 0.1% formic acid. An internal standard mix is also prepared with deuterated bile acid standards with each having a final concentration of 100 µM.

### 4.3. Mice and Human Samples

All mouse experiments were conducted in accordance with FELASA guidelines [36] and were approved by the Animal Welfare Officers of the University Medical Center Hamburg-Eppendorf (UKE) as well as the Behörde für Gesundheit und Verbraucherschutz Hamburg (animal protocol N008/2020, approved on 18 March 2020). Cyp2c70 knockout mice and corresponding C57BL/6J wild type control mice were used in this study. All animals were bred and maintained in the animal facility of the University Medical Center Hamburg-Eppendorf. Mice were housed under standard conditions at room temperature with a 12 h light/12 h dark cycle and had ad libitum access to food and water. Age-matched female mice aged 13–15 months were fed standard laboratory chow diet ad libitum (19.1% protein, 4% fat, 6% fiber; Altromin Spezialfutter GmbH & Co. KG, Lage, Germany). Mice were anesthetized by CO_2_ inhalation, followed by cervical dislocation. Livers were harvested immediately and snap-frozen in liquid nitrogen.

Human liver samples were collected from patients undergoing bariatric surgery at the Department of General, Visceral and Thoracic Surgery, University Medical Center Hamburg-Eppendorf. Liver biopsies were immediately placed on ice and subsequently snap-frozen in liquid nitrogen and stored in liquid nitrogen or at −80 °C. All participants signed an informed consent; the study was approved by the Ethics Committee of the Hamburg Chamber of Physicians (PV4889) and conducted in accordance with the declaration of Helsinki.

### 4.4. Laser Ablation Setup and Workflow

**Figure 6:**
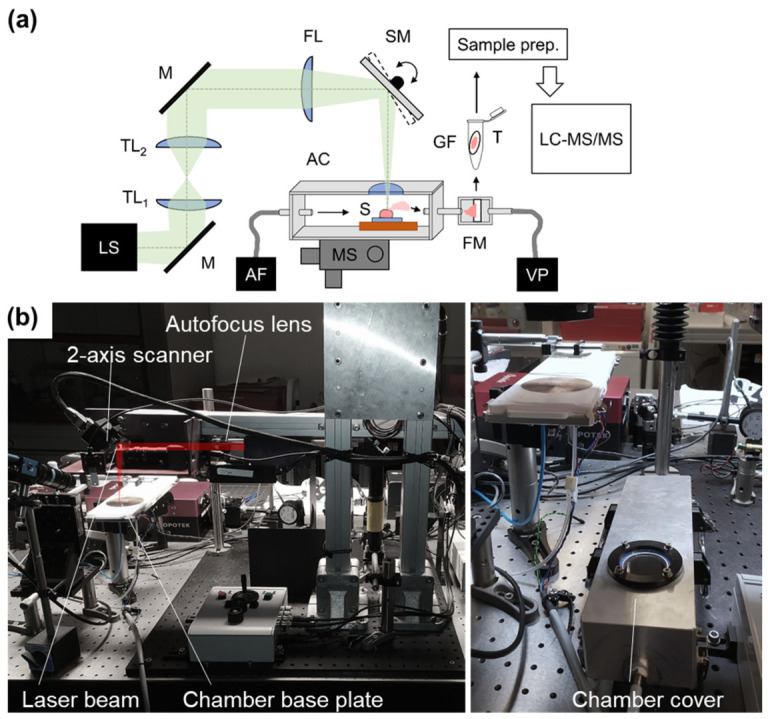
(**a**) Schematic representation of the NIRL setup and workflow from ablation to sample preparation for LC-MS/MS. (**b**) Real-world picture of the NIRL highlighting the laser beam, 2-axis scanner, autofocus lens, chamber base plate and chamber cover. Green: divergent beam; LS: NIRL laser system; M: silver mirror; TL1, TL2: telescope lenses; FL: focusing lens (of 150 mm focal length); SM: two-axis scanning mirror; AC: ablation chamber; S: scanning of the sample; MS: manual xyz stage; AF: air flow with filter; FM: filter mount; VP: vacuum pump; GF; glass fiber filter; and T: transfer tube.

Freshly frozen liver tissue was placed on a glass slide within an ablation chamber and ablated on a cooling copper metal block with temperature maintained at −10 °C. The NIRL (Opolette SE 2731, Opotek, LLC, Carlsbad, CA, USA) was tuned to the wavelength of 2940 nm and focused down to a diameter of 125 µm, delivering a pulse energy of 1.3 mJ at the sample position. A scanning mirror, synchronized to the laser pulses applied a meander scan pattern measuring 2 mm × 2 mm with 24 repetitions, resulted in a cubic ablation volume. With the help of a vacuum pump, the generated liver aerosol was then collected on a glass fiber filter paper (GF50 grade, glass fiber filter without binders, Hahnemühle FineArt, Dassel, Germany) with a diameter of approximately 10 mm. The laser setup depicting the laser path, ablation chamber and sample stage setup is shown in Figure 6a, where a real-world image of the NIRL is pictured in Figure 6b.

### 4.5. Determination of Ablated Tissue Volumes

The ablated tissue volume was determined utilizing a spectral domain optical coherence tomography system (TEL221PSC2-SP1, Thorlabs, Newton, NJ, USA) with a central wavelength of 1300 nm. The imaging volume was set to 6.0 × 6.0 × 2.65 mm^3^ with a voxel size of 8.23 × 8.23 × 3.45 µm^3^ in air. Based on image stacks before and after ablation, the ablated volume was manually segmented and quantified in the open source software ITK-SNAP version 3.8.0 [37]. The dimensions were determined on basis of central brightness scans (B-scans), as shown in Figure 2 in the results section.

### 4.6. Bile Acid Extraction

To freshly frozen liver tissues, 1 mL of butylated hydroxytoluene in methanol (1 g/L) was added in a 2 mL Eppendorf tube. Next, 10 µL of internal standard mix and one metal ball was added prior to tissue homogenization with the TissueLyser (TissueLyser III, Qiagen, Hilden, Germany) for 3.5 min at a frequency of 30 Hz. The homogenate was then centrifuged for 10 min at 10,000× *g* and 4 °C. The supernatant was transferred onto a PTFE filter using a 1 mL syringe for filtration into a new tube. The filtrate was then evaporated in a vacuum centrifuge and the bile acids re-suspended in 100 µL of solution containing methanol/acetonitrile (1/3, *v*/*v*), 20 mM ammonium acetate and 0.1% formic acid.

To the glass fiber filter paper with the condensed NIRL ablated aerosol, 1 mL of butylated hydroxytoluene in methanol (1 g/L) was added in a 2 mL Eppendorf tube. Next, 5 µL of internal standard mix and one metal ball was added and vortexed for 1 min. The vial was then centrifuged for 10 min at 10,000× *g* and 4 °C. The supernatant transferred onto a PTFE filter using a 1 mL syringe for filtration into a new tube. The filtrate was evaporated in a vacuum centrifuge and the bile acids re-suspended in 50 µL of solution containing methanol/acetonitrile (1/3, *v*/*v*), 20 mM ammonium acetate and 0.1% formic acid. Of note, the extraction efficiency of the bile acids from the glass fiber filter paper was not experimentally validated.

### 4.7. LC-MS/MS Data Acquisition

In this study, LC-MS/MS was conducted using a Shimadzu Nexera X2 HPLC system (Shimadzu, Tokyo, Japan) connected to a Qtrap 5500 mass spectrometry System (SCIEX, Darmstadt, Germany). A Kinetex C18 column (100 Å, 150 mm × 2.1 mm i.d., Phenomenex, Torrance, CA, USA) was used to separate the bile acids. Each sample was run twice for acquisition in the MRM mode with both positive and negative electrospray ionization techniques. The mass spectrometry method used is established and validated by Wegner et al. [38]. For positive acquisition, the bile acids were eluted in a gradient elution fashion starting with a pump B concentration at 30%. It was then increased to 50% in 7 min and to 100% over the next 8 min. It was held constant at 100% for 10 min and brought back to 30% in 2 min followed by 6 min of re-equilibration. The eluted analytes were then positively ionized in the mass spectrometer and the data acquired in scheduled MRM mode, where the mass transitions between precursor and product ions for each analyte and its retention time was taken into consideration. The negative method was done similarly in gradient elution with pump B initially at 30% for a minute and was increased to 60% in another minute. It was then increased to 80% in 4 min and held constant for 3 min. It was then increased to 100% over 3 min and held there for 4 min, brought down to 40% in 2 min and left to re-equilibrate for 3 min. Both methods used a mobile phase A which contained 0.1% formic acid in MS water and B containing methanol/acetonitrile (1/3, *v*/*v*), 20 mM ammonium acetate and 0.1% formic acid. The MRM transitions and retention times used for both methods can be seen in Supplementary Tables S5 and S6. The entire LC-MS/MS system was controlled using Analyst 1.6.1 software (AB Sciex, Concord, ON, Canada). Data analysis was conducted using Multiquant 3.0.3 and the SCIEX OS version 2.0 software (AB Sciex, Concord, ON, Canada).

### 4.8. Statistical Analysis

All data present in the manuscript as means are presented as means ± standard deviation. Group sizes are indicated in the figure legends. For the comparison of two groups, statistical analysis was performed using an unpaired Student’s *t* test. GraphPad Prism 11.0.1 (GraphPad Software, Inc., La Jolla, CA, USA) was used for statistical calculations and *p* values below 0.05 were considered statistically significant.

## Data Availability

Mass spectrometric raw data was automatically processed by the Mutiquant software 3.0.3. The corresponding quantitative data can be found in Supplementary Tables S3–S5.

## Supplementary Materials

The following supporting information can be downloaded at: https://www.mdpi.com/article/10.3390/ijms27104572/s1.

## Abbreviations

The following abbreviations are used in this manuscript:

NIRL	nanosecond infrared laser
OCT	optical coherence tomography imaging
LC–MS	liquid chromatography mass spectrometry
LCA	lithocholic acid
TLCA-3S	taurolithocholic acid-3 sufate
TLCA	taurolithocholic acid
CA	cholic acid
UCA	ursocholic aicd
3-oxo-DCA	3-oxo-deoxycholic acid
7-oxo-CA	7-ketodeoxycholic acid
TUDCA	tauroursodeoxycholic acid
THDCA	taurohyodeoxycholic acid
TCA	taurocholic acid
GCA	glycocholic acid
TCDCA	taurochenodeoxycholic acid
TDCA	taurodeoxycholic acid
GCDCA	glycochenodeoxycholic acid
GDCA	glycodeoxycholic acid
CDCA	chenodeoxycholic acid
GLCA	glycolithocholic acid
DCA	deoxycholic acid
5CholA	5-cholenic acid
isoDCA	isodeoxycholic acid
isoalloLCA	isoallolithochlic acid
isoLCA	isolithocholic acid
wMCA	omega muricholic acid
aMCA	alpha muricholic acid
bMCA	beta muricholic acid
TwMCA	tauro-omega muricholic acid
TaMCA	tauro-alpha muricholic acid
TbMCA	tauro-beta muricholic acid
