# Supplementary material for: Nanosecond Infrared Laser Sampling of Mouse and Human Liver Tissues for LC-MS/MS Analysis of Bile Acids
Source: Int J Mol Sci. 2026 May 20;27(10):4572. doi: 10.3390/ijms27104572 (PMC13207702; doi:10.3390/ijms27104572)
Supplement: Supplementary file 1 [file ijms-27-04572-s001.zip › Supplement Figure 1 Fuh et al.pptx]

## Slide 1
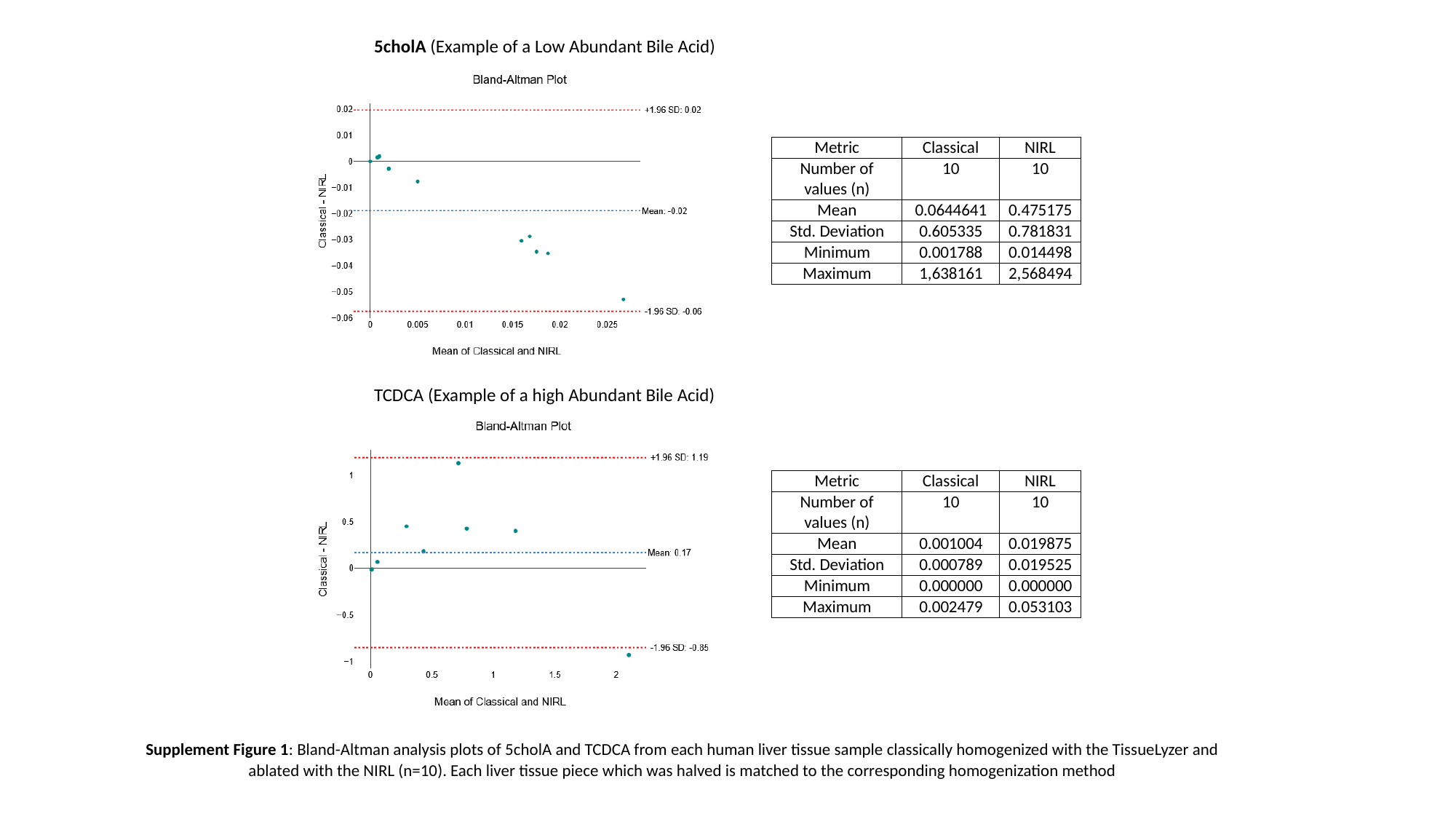

5cholA (Example of a Low Abundant Bile Acid)
TCDCA (Example of a high Abundant Bile Acid)
Supplement Figure 1: Bland-Altman analysis plots of 5cholA and TCDCA from each human liver tissue sample classically homogenized with the TissueLyzer and ablated with the NIRL (n=10). Each liver tissue piece which was halved is matched to the corresponding homogenization method
